# Metagenomics reveals differences in microbial composition and metabolic functions in the rumen of dairy cows with different residual feed intake

**DOI:** 10.1186/s42523-022-00170-3

**Published:** 2022-03-08

**Authors:** Yunyi Xie, Huizeng Sun, Mingyuan Xue, Jianxin Liu

**Affiliations:** 1 Institute of Dairy Science, College of Animal Sciences, Zhejiang University, Hangzhou, 310058 China; 2 Ministry of Education Key Laboratory of Molecular Animal Nutrition, Zhejiang University, Hangzhou, 310058 China

**Keywords:** Dairy cattle, Metagenomics, Residual feed intake, Rumen microbiota

## Abstract

**Background:**

Rumen microbial composition and functions have vital roles in feed digestion and fermentation and are linked to feed efficiency in cattle. This study selected Holstein cows, which are high in both milk protein content and milk yield, to analyse the relationship between the rumen microbiota and residual feed intake (RFI). Eighteen multiparous lactating cows were divided into low RFI (LRFI, high efficiency, n = 9) and high RFI (HRFI, low efficiency, n = 9) groups to investigate the differences in microbial composition and functions.

**Results:**

The relative abundances of butyrate producers, including the *Clostridium, Butyrivibrio, Eubacterium* and *Blautia* genera, were higher in HRFI cows than in LRFI cows (*P* < 0.05). Four carbohydrate metabolic pathways (glycolysis/gluconeogenesis, pentose phosphate pathway, fructose and mannose metabolism, and butanoate metabolism) and one energy metabolism pathway (methane metabolism), were more abundant in HRFI animals (*P* < 0.05). Quorum sensing and DNA replication pathways were more abundant in HRFI cows. For CAZyme profiles, 14 out of 19 genes encoding carbohydrates-deconstructing enzymes were more abundant in HRFI cows (*P* < 0.05). Seven *Lachnospiraceae* species associated with carbohydrate metabolism and quorum sensing may contribute to the difference in feed efficiency. Moreover, the LRFI cows had lower abundances of *Methanosphaera* (*P* < 0.01), *Methanobrevibacter ruminantium* (*P* = 0.09) and methanogenesis functions (*P* = 0.04).

**Conclusions:**

The rumen microbiota of low-efficiency cows has stronger abilities to degrade carbohydrates and produce methane, and quorum sensing pathways could also be associated with differences in feed efficiency. This study provides a deeper understanding of the microbial ecology of dairy cows with different feed efficiencies and highlights the possibility of modulating the rumen microbiome or microbial functions to improve the feed efficiency of dairy cows.

**Supplementary Information:**

The online version contains supplementary material available at 10.1186/s42523-022-00170-3.

## Introduction

Feed efficiency is an important trait in the dairy industry since feed inputs account for 40–60% of the total variable costs in dairy farms [1]. Improving the feed efficiency of dairy cows has profound significance, as it is expected to decrease the total feed cost and reduce greenhouse gas emissions and urinary nitrogen excretion. Additionally, there are various methods to evaluate feed efficiency, among which residual feed intake (RFI) is one of the most common measurements in cattle [2, 3]. The RFI is defined as the difference between the actual and predicted dry matter intake (DMI) for an animal. A low or negative RFI value represents high efficiency, while a high RFI value represents low efficiency. The RFI is independent of the level of production, and animals with lower values are deemed more efficient [4].

Physiological processes have been identified as important factors contributing to the differences in RFI among dairy cows, including feeding behaviour, activity, digestibility, and rumen-temperature variables [5, 6]. Several studies have reported that rumen fermentation variables and nutrient absorption affect the RFI of cattle [7, 8]. Rumen fermentation is largely mediated by diverse rumen microbes to produce the major products volatile fatty acids (VFAs), which can provide greater than 70% of the energy requirement for ruminants [9]. Therefore, the rumen microbiome plays a vital role in variations in RFI.

Previous studies have reported differences in the rumen microbial composition between efficient and inefficient beef cattle [10, 11]. McGovern et al. [12] found that the abundances of *Lactobacillales* and *Ruminobacter* genera were associated with RFI. Lower abundances of *Methanosphaera stadtmanae* and *Methanobrevibacter sp.* has been identified in high RFI animals [13]. Most of these studies used beef cattle or growing heifers, but there are distinct differences between dairy and beef cattle. Limited studies have shown a correlation between rumen microbial ecology and RFI in lactating dairy cattle [13]. However, a high concentrate diet (70% concentrate) may lead to abnormal ruminal microbial metabolism and further affect the rumen microbiota. Thus, it is important to examine the relationship between RFI and the microbiome in cows fed an optimal forage-to-concentrate ratio. Furthermore, a previous study did not address the impact of differences in individual performance (e.g., milk protein yield) on microorganisms [13]. Xue et al. [14] revealed the differences in the rumen microbiome and its metabolites between individual cows with different milk protein yields and indicated a correlation between rumen ecology and host performance. In the present study, we profiled the rumen microbial composition and functions in dairy cows with relatively high milk yield and milk protein content that had divergent RFI to uncover the relationship between RFI and microbiome components. The results will provide a better understanding of the rumen microbiome and its impact on the RFI of lactating dairy cattle.

## Results

### Characterization of phenotypes

The low RFI (LRFI) cows consumed 2.43 kg dry matter/d less than their high RFI (HRFI) counterparts. The ratios of milk yield to DMI (*P* < 0.01) and of energy-corrected milk to DMI (*P* = 0.03) were greater in LRFI group than in HRFI group (Additional file 1: Table S1). Although the percentage of milk protein was significantly lower (*P* = 0.04) in the LRFI group, no significant differences (*P* > 0.10) were detected in the yields of milk and milk protein between the two groups.

The concentrations of total VFAs (*P* = 0.08), acetate (*P* = 0.10), butyrate (*P* = 0.10), valerate (*P* = 0.08) and isovalerate (*P* = 0.09) tended to be lower in the LRFI group (*P* < 0.10, Table 1). However, the molar proportion of propionate tended to be higher in LRFI cows (24.0 vs. 22.7, *P* = 0.09), and the molar proportions of isobutyrate (0.90 vs. 0.74, *P* = 0.05) and isovalerate (1.50 vs. 1.23, *P* < 0.01) were significantly greater in LRFI cows than HRFI cows. Methane (CH_4_) production was higher in HRFI cows than in LRFI cows (*P* = 0.04).

**Table 1:** Fermentation variables and methane production in the rumen of dairy cows with high and low residual feed intake (RFI)

Items	High RFI	Low RFI	SEM	*P* value
pH	6.40	6.46	0.06	0.57
Volatile fatty acid, mM				
Total	105	96.7	3.25	0.08
Acetate	66.3	59.4	2.78	0.10
Propionate	23.7	23.5	0.82	0.88
Butyrate	11.7	10.2	0.60	0.10
Isobutyrate	0.76	0.86	0.04	0.12
Valerate	1.52	1.32	0.08	0.08
Isovalerate	1.27	1.45	0.07	0.09
Molar proportion, mM/100 mM				
Acetate	62.8	61.6	1.15	0.47
Propionate	22.7	24.0	0.53	0.09
Butyrate	11.0	10.5	0.28	0.30
Isobutyrate	0.74	0.90	0.05	0.05
Valerate	1.44	1.38	0.06	0.43
Isovalerate	1.23	1.50	0.06	< 0.01
Acetate: propionate	2.90	2.63	0.19	0.30
Methane^a^, g/d	533	506	8.50	0.04

^a^Methane was predicted by the following equation: CH_4_ (g/d) = [3.23 (± 1.12) + 0.81 (± 0.086) × DMI (kg/d)] × 18.03 [36], where DMI is the dry matter intake

### The rumen metagenome profile

A total of 909,094,550 raw reads were obtained from the 18 rumen digesta samples, with an average of 50,505,253 ± 928,365 (SEM) raw reads per sample. After quality control and host gene removal, 901,313,532 clean reads, with 50,072,974 ± 921,079 raw reads per sample, were retained (Additional file 1: Table S2). Multiple megahit assembly of the raw sequencing reads resulted in 11,670,011 contigs, with an average of 648,334 ± 44,632 contigs per sample. At the domain level, the rumen metagenome consisted of 94.84% bacteria, 3.10% eukaryotes, 1.33% archaea, and 0.51% viruses (Additional file 1: Fig. S1).

### Differences in microbial community compositions between LRFI and HRFI cows

**Fig. 1:**
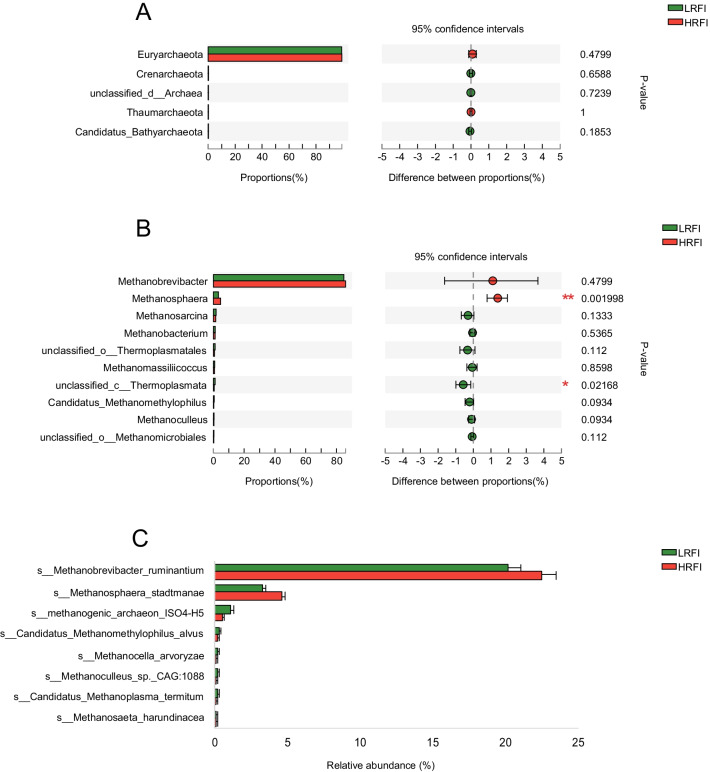
Comparison of archaeal phyla, genera and species between the cows with high (HRFI) and low residual feed intake (LRFI). The difference of archaeal phyla (**A**) and genera (**B**) were tested by Wilcoxon rank-sum test, *P < 0.05, **P < 0.01, (**C**) Significantly different archaeal species. Significant differences were tested by Linear discriminant analysis effect size (LEfSe) analysis, with linear discriminant analysis (LDA) score of > 2 and *P* value of < 0.05

The permutational multivariate analysis of variance (PERMANOVA) showed no differences between HRFI and LRFI groups in bacteria (*P* = 0.17), eukaryotes (*P* = 0.25) archaea (*P* = 0.15) or viruses (*P* = 0.13, Additional file 1: Table S3). The principal coordinate analysis (PCoA) did not show clear separation between the HRFI and LRFI groups based on archaeal species (Additional file 1: Fig. S2a). Meanwhile, no significant difference was found between the HRFI and LRFI groups in archaeal phyla. However, the abundance of *Methanosphaera* was higher in HRFI group (4.66 vs. 3.28, *P* < 0.01). The relative abundance of *Methanobrevibacter ruminantium*, the most abundant archaeal species, tended to be higher in HRFI cows than in LRFI cows (22.45 vs. 20.14, *P* = 0.09, Fig. 1).

**Fig. 2:**
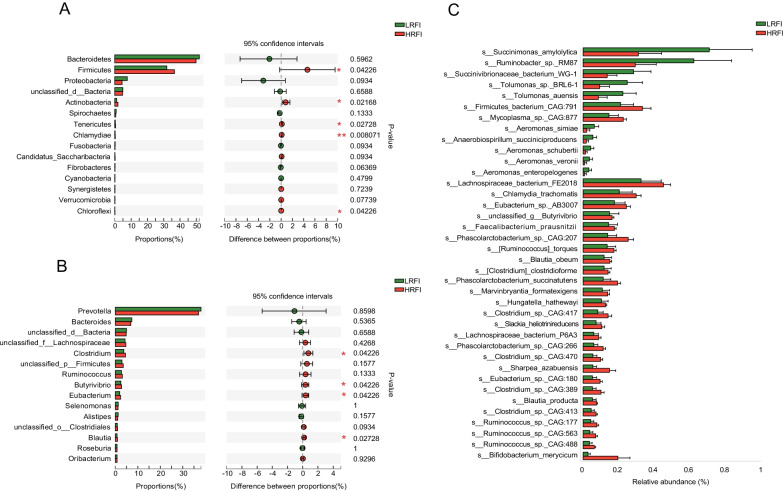
Comparison of bacterial phyla, genera and species between the cows with high (HRFI) and low residual feed intake (LRFI). The difference of bacterial phyla (**A**) and genera (**B**) were tested by Wilcoxon rank-sum test, *P < 0.05, **P < 0.01. (**C**) Significant different bacterial species. Significant differences were tested by linear discriminant analysis effect size (LEfSe) analysis, with linear discriminant analysis (LDA) score of > 2 and *P* value of < 0.05

The PCoA of bacterial species showed no clear separation between the HRFI and LRFI groups (Additional file 1: Fig. S2b). At the phylum level, compared with LRFI group, the relative abundance of *Firmicutes* was significantly greater in HRFI group (36.57 vs. 31.91, *P* = 0.04), and *Proteobacteria* tended to be lower in the HRFI cows than in LRFI cows (4.45 vs. 7.61., *P* = 0.09, Fig. 2A). At the genus level, the abundances of *Clostridium, Butyrivibrio, Eubacterium* and *Blautia* were significantly higher in HRFI cows (*P* < 0.05, Fig. 2B). At the species level, 28 species, including 24 *Firmicutes sp.,* showed significantly higher abundances in HRFI group (LDA > 2, *P* < 0.05), while 10 species were more abundant in the LRFI group (LDA > 2, *P* < 0.05, Fig. 2C).

### Differences in functions of the rumen microbiome between LRFI and HRFI cows

For Kyoto Encyclopedia of Genes and Genomes (KEGG) profiles, a total of 206 endogenous level-3 pathways were observed in our study. These pathways belonged to 4 level-1 KEGG functional categories, including “metabolism” (69.67 ± 0.47%), “genetic information processing” (15.77 ± 0.07%), “cellular processes” (7.38 ± 0.02%) and “environment information processing” (7.18 ± 0.02%). At the second level of KEGG pathways, a total of 24 functional categories were identified. The categories “carbohydrate metabolism” (16.54 ± 0.14%), “global and overview maps” (11.62 ± 0.13%), “amino acid metabolism” (10.25 ± 0.10%), “replication and repair” (8.07 ± 0.10%), and “nucleotide metabolism” (10.25 ± 0.10%) were the most abundant functions.

**Fig. 3:**
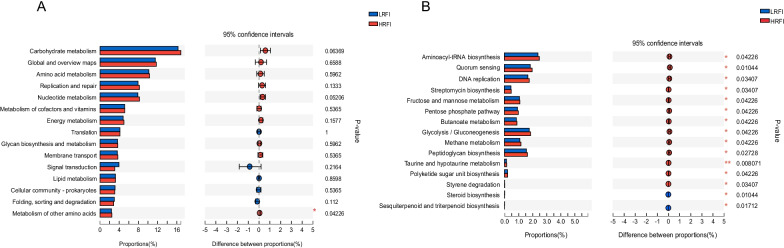
Differential KEGG functions between the cows with high (HRFI) and low residual feed intake (LRFI). **A** KEGG functions on level 2 tested by Wilcoxon rank-sum test. **B** Significantly different KEGG functions on level 3 tested by Wilcoxon rank-sum test; * *P* < 0.05, ** *P* < 0.01

The PCoA indicated no separation in functional potential between the two feed efficiency cohorts (Additional file 1: Fig. S3a). At KEGG level 2, the category “carbohydrate metabolism” tended to be higher in the HRFI animals than in the LRFI animals (*P* = 0.06, Fig. 3A). At KEGG level 3, 13 pathways, including 10 “metabolism” pathways, 2 “genetic information processing” pathways, and 1 “cellular processes” pathway, were upregulated in HRFI cows (*P* < 0.05; Fig. 3B), and two KEGG pathways were significantly enriched in LRFI cows (*P* < 0.05; Fig. 3B). Within the category “carbohydrate metabolism”, “ko00010: Glycolysis/gluconeogenesis”, “ko00030: pentose phosphate pathway”, “ko00051: fructose and mannose metabolism”, and “ko00650: butanoate metabolism” were significantly enriched in the HRFI cows (*P* < 0.05; Fig. 3B). For the category “energy metabolism”, only “ko00680: methane metabolism” was enriched in HRFI cows (*P* = 0.04, Fig. 3B). For the other highly abundant biological processes, “DNA replication” and “quorum sensing” were higher in the HRFI group (*P* < 0.05; Fig. 3). However, “ko00100: steroid biosynthesis” and “ko00909: sesquiterpenoid and triterpenoid biosynthesis” were significantly enriched in LRFI cows (*P* < 0.05; Fig. 3B).

### CAZyme functions of the rumen microbiome between LRFI and HRFI cows

In terms of CAZyme profiles, 199 genes were detected, including 97 glycoside hydrolases (GHs), 42 glycosyltransferases (GTs), 31 carbohydrate-binding modules (CBMs), 14 carbohydrate esterases (CEs), 10 polysaccharide lyases (PLs), and 5 auxiliary activities (AAs). Among them, GT2 (6.99% ± 0.15%), GH2 (6.33% ± 0.08%), and GT4 (3.85% ± 0.05%) were the most abundant CAZymes in the rumen of these cows.

**Fig. 4:**
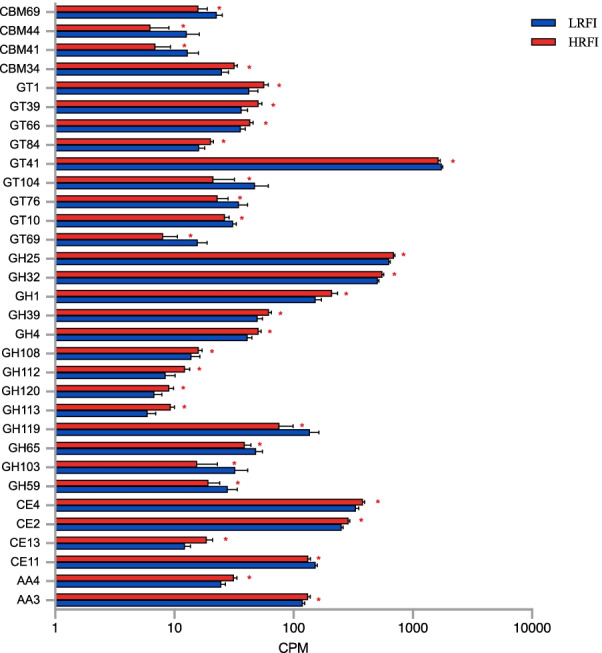
Significantly different CAZyme functions between the cows with high (HRFI) and low residual feed intake (LRFI). AA: Auxiliary Activities; CBM: Carbohydrate-Binding Modules; CE: Carbohydrate Esterases; GH: Glycoside Hydrolases. GT: Glycosyl Transferases. * *P* < 0.05

No obvious separation was observed for the CAZyme profiles between feed efficiency groups based on PCoA (Additional file 1: Fig. S3b). We then compared the relative abundance of CAZymes, and 19 differentially abundant CAZymes related to deconstructing carbohydrates (GH, CE, PL, AA, and CBM) were identified between the two RFI groups. Only five CAZymes (4 GHs, 1 CE) were more abundant in LRFI animals, while 14 CAZymes (9 GHs, 3 CEs, and 2 AAs) were enriched in HRFI animals. Among the GTs, five CAZymes were more abundant in the LRFI group, while four CAZymes were enriched in HRFI cows. Regarding the CBMs, three CAZymes were more abundant in the LRFI group, while only one was enriched in the HRFI group (*P* < 0.05; Fig. 4).

### Relationships between microbial species and functions

**Fig. 5:**
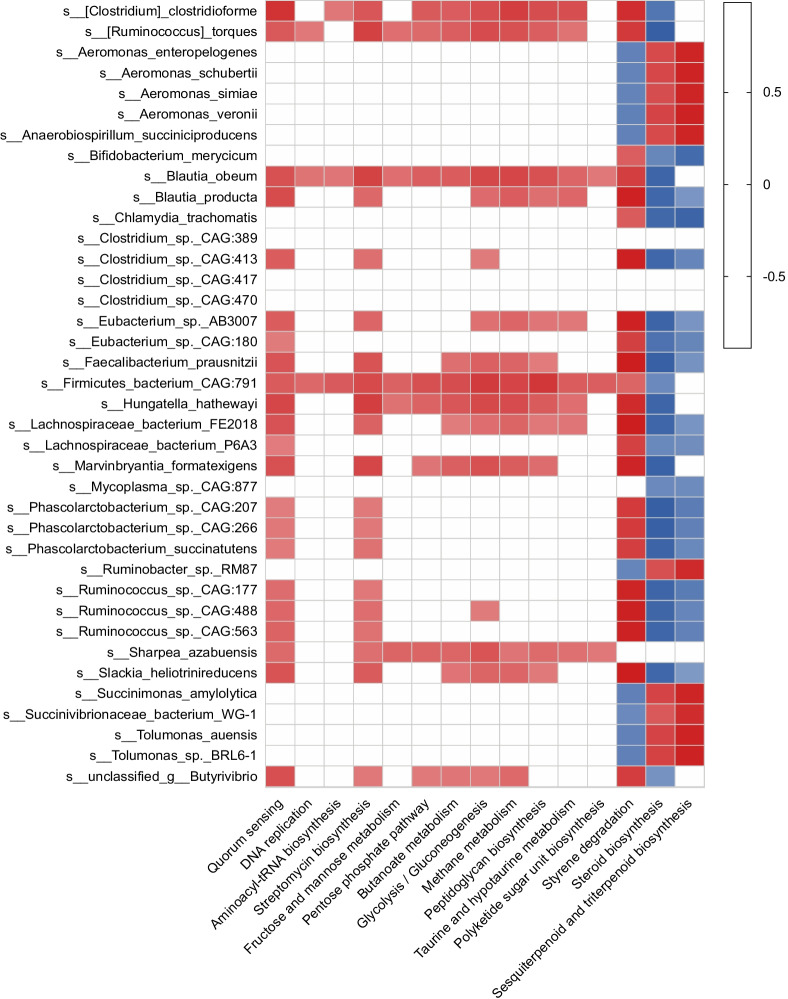
Correlation patterns showing associations between differential microbial species and metabolic pathways. Correlation analyses were conducted using Spearman’s rank correlation. Only strong (correlation coefficient R > 0.5 or < − 0.5) and significant (*P* < 0.05) correlations were selected to be displayed

*Spearman’s* rank correlation was constructed between differential microbial species and metabolic pathways. A total of 38 species showed significant relationships with 15 differentially abundant pathways (R > 0.50 and *P* < 0.05, Fig. 5), and 22 species had positive relationships with “quorum sensing” pathway (R > 0.50 and *P* < 0.05. Figure 5). Among these 22 species, 15 also had a positive relationship with “carbohydrate metabolism” pathways (ko00010, ko00030, ko00051, and ko00650, Fig. 5). Among these 19 species, 7 belonged to the family *Lachnospiraceae,* including *Ruminococcus torques*, *Clostridium clostridioforme*, *Blautia obeum*, *B. producta*, *L. bacterium FE2018, Marvinbryantia formatexigens* and an unclassified* Butyrivibrio sp.* (Fig. 5).

**Fig. 6:**
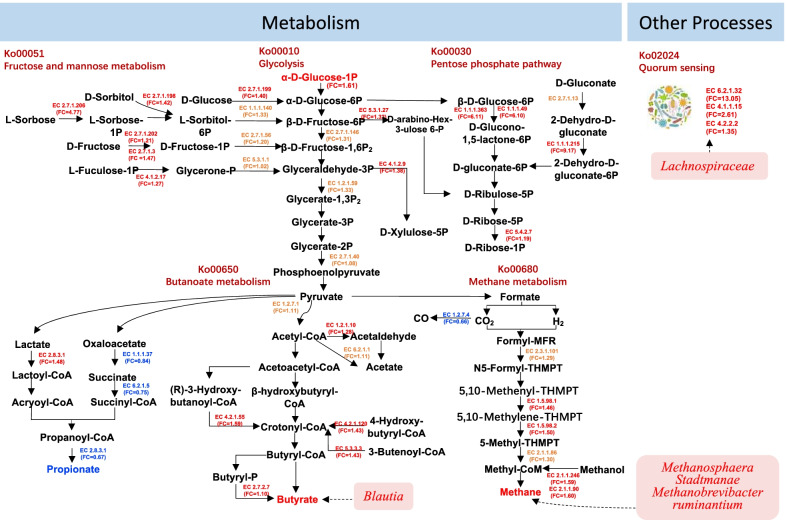
Microbial functions and species involved in carbohydrate metabolism, energy metabolism and other processes in the rumen of the cows with high (HRFI) and low residual feed intake (LRFI). Among the genes encoding enzymes involved in the fructose and mannose metabolism pathway, 8 genes tended to be more abundant in the HRFI cows. In the glycolysis pathway, the abundance of 4 genes tended to be higher in the HRFI cows (*P* < 0.10), and 1 gene was more abundant in the HRFI cows (*P* = 0.03). In the pentose phosphate pathway, 2 genes tended to be more abundant in the HRFI group (*P* < 0.10), and 5 genes were enriched in the HRFI group (*P* < 0.05). Three genes involved in quorum sensing pathways, and 6 genes involved in methane metabolism were significantly enriched in the HRFI cows. In the butyrate metabolism pathway, 4 genes tended to be more abundant in HRFI cows (*P* < 0.10), and 3 genes related to butyrate production were significantly enriched in HRFI cows (*P* < 0.05). The blue text represents the genes or metabolites enriched in HRFI cows, and the red (orange) text indicates the genes or metabolites enriched or tended to be enriched in LRFI cows. The text in brackets under the gene name is HRFI/LRFI fold change of gene. The text over the arrow is the gene code. Inside the pink rectangles are the microorganisms that play an important role in the pathway

Microbial functions and species related to carbohydrate metabolism, energy metabolism and other processes in the rumen of the animals are summarized in Fig. 6. Among the genes encoding enzymes involved in the fructose and mannose metabolism pathway, 8 genes, EC 1.1.1.140, EC 2.7.1.198, EC 2.7.1.202, EC 2.7.1.206, EC 2.7.1.3, EC 2.7.1.56, EC 4.1.2.17 and EC 5.3.1.1, tended to be more abundant in the HRFI cows (*P* < 0.10). In the glycolysis pathway, the abundance of EC 1.2.1.59, EC 2.7.1.146 and EC 2.7.1.40 tended to be higher in the HRFI cows (*P* < 0.10), and EC 2.7.1.199 was more abundant in the HRFI cows (*P* = 0.03). In the pentose phosphate pathway, two genes (EC 2.7.1.13 and EC 5.3.1.27) tended to be more abundant in the HRFI group (*P* < 0.10), and 5 genes (EC 1.1.1.215, EC 1.1.1.363, EC 1.1.1.49, EC 4.1.2.9 and EC 5.4.2.7) were enriched in the HRFI group (*P* < 0.05). Three genes (EC 4.1.1.15, EC 4.2.2.2 and EC 6.2.1.32) involved in quorum sensing pathways, and 6 genes (EC 1.5.98.1, EC 1.5.98.2, EC 2.1.1.248, EC 2.1.1.86, EC 2.1.1.90 and EC 2.3.1.101) involved in methane metabolism were significantly enriched in the HRFI group (*P* < 0.05). In the butyrate metabolism pathway, four genes (EC 1.2.1.10, EC 1.2.7.1, EC 2.7.2.7 and EC 6.2.1.1) tended to be more abundant in HRFI cows (*P* < 0.10), and 3 genes (EC 4.2.1.120, EC 4.2.1.55, EC 5.3.3.3) related to butyrate production were significantly enriched in HRFI cows (*P* < 0.05). However, in the succinate pathway for propionate production, three genes encoding enzymes (EC 1.1.1.37, EC 6.2.1.5, EC 2.8.3.1) were more abundant in the LRFI cows.

## Discussion

Improvement in feed efficiency could greatly reduce feed costs and increase profits. In our study, LRFI cows with similar milk production consumed 2.50 kg less dry matter per day than the HRFI cows did, which is consistent with previous studies [15, 16]. The conversion of feed to VFAs is dependent on rumen microbes, and the rumen microbiome plays an important role in determining an animal’s RFI [17, 18]. In our study, the microbial differences at the taxonomic and functional levels between the two RFI groups explain the underlying mechanisms.

A greater amount of bacterial species (28 vs. 10) were more abundant in HRFI cows than in LRFI cows, which is in line with previous findings showing that HRFI animals tended to have higher microbiome richness [13]. Our results found higher abundances of *Clostridium, Butyrivibrio, Eubacterium* and *Blautia* in HRFI cows. *Clostridium* species ferment carbohydrates to form hydrogen and butyrate [19]. The genus *Butyrivibrio* plays an important role in hemicellulose and pectin breakdown in the rumen, and the end product of this fermentation is butyrate [20, 21]. The genera *Eubacterium* and *blautia* possess the capability to produce butyrate [22]. Moreover, most of the species that abundances were higher in HRFI cows were butyrate producers and showed positive associations with the “ko00650: butanoate metabolism” pathway, suggesting their important roles in butyrate biosynthesis. Indeed, we detected a trend towards higher butyrate concentrations in HRFI cows.

*Methanosphaera* is a methanogenic archaeon that utilizes methyl group compounds to generate methane [23]. In the rumen, methanol is a product of pectin hydrolysis by protozoa and esterase activity of bacteria [24]. The higher DMI in HRFI cows means more substrates available for fermentation in the rumen. The greater potential for increased availability of methanol is associated with the higher abundance of *Methanosphaera* in HRFI group in the current study, suggesting the importance of *Methanosphaera* in affecting host feed efficiency. The higher abundance of *Methanobrevibacter ruminantium* in HRFI cows was consistent with the study of Shabat et al. [13]. These results suggest that HRFI cows may produce more methane, leading to energy waste and lower efficiency. Moreover, methane metabolism and KO genes related to methanogenesis were more abundant in HRFI cows. In line with our results, previous studies also reported a higher abundance of methanogenesis pathways in HRFI cows [13]. Methane emissions cause a significant loss of dietary energy and reduce the efficiency of animals [23]. A higher abundance of KO genes related to propionate production and a higher tendency of the molar proportion of propionate in LRFI cows indicates that the rumen microbiomes of LRFI cows are more likely to generate substances with greater usable energy values. As mentioned above, we found a lower tendency of butyrate concentrations and higher tendency of the molar proportion of propionate in LRFI cows. Moss et al. [25] reported that formation of butyrate results in production of additional methanogenic substrates (formate and H_2_), while propionate formation is involved in hydrogen utilisation, which can be considered as a competitive pathway for hydrogen use in the rumen. Thus, the lower butyrate concentrations and higher molar proportion of propionate in LRFI cows may decrease methanogenesis by competing for hydrogen.

The functional genes of the rumen microbiome identified by metagenomics provide a way to evaluate the functions of rumen microorganisms [26, 27]. In a recent study, Xue et al. [14] found that rumen microbial functions (21.56%) make a greater contribution to milk protein yield than rumen microbial composition (17.81%). These findings suggest that the rumen microbial function may reflect the mechanism by which microorganisms influence phenotypes. Previous studies used metagenomics [13] or metatranscriptomics [26] to investigate the functional profiles of rumen microbiomes in cows with different RFI. A metagenomic study using rumen samples from beef cattle reported a higher number of KEGG pathways enriched in HRFI cattle [26], consistent with the results in our study. Shabat et al. [13] investigated the linkages between the rumen microbiome and host efficiency in dairy cows with different RFI and revealed less diverse metabolic pathways in efficient cows, suggesting that efficient microbiomes are more specialized to meet the energetic need of ruminants. Additionally, we found that the KEGG functions of carbohydrate degradation, including “glycolysis”, “pentose phosphate pathway” and “fructose and mannose metabolism”, were enriched in the rumen of HRFI animals. These results indicate that HRFI microbiome may generate more diverse products such as pyruvate, acetyl-CoA and hydrogen. In the current study, HRFI cows had a higher number of CAZyme-encoding genes. Similar results were reported at the RNA level in beef cattle in a metatranscriptomics study [26]. The higher abundance of KO genes related to carbohydrate degradation in HRFI cows indicates higher activities of degrading complex substrates in inefficient cows. Most species showing positive relationships with carbohydrate metabolism belonged to the *Lachnospiraceae* family. Many members of the family *Lachnospiraceae* have cellulolytic activity and are related to butyrate production [28, 29]. In the current study, we found increased butyrate metabolism in HRFI cows. Therefore, the *Lachnospiraceae* species may play an essential role in carbohydrate metabolism and further affect feed efficiency in dairy cows. However, it is unclear whether the rumen microbiota is either the causes or results of cattle feed efficiency. Thus, our results warrant further research to fully elucidate the relationship between the alteration in microbiota and host functional changes.

The higher activity of rumen microbiomes in HRFI animals could be attributed to their higher feed intake. Pathak [30] reported a strong positive correlation of microbial activities with feed intake because more available substrates and nutrients from animals with higher feed intake were provided for microbial growth. Indeed, the higher abundance of the DNA replication pathway in our current study provides further evidence for the greater microbial growth rate in HRFI cows. The higher microbial growth rate may result in higher population density in bacteria. Enrichment of the quorum sensing pathway in HRFI cows has not been reported previously. In a recent study, some of the bacterial species in rumen were identified the existence of *luxS* genes, and these species showed positive relationships with quorum sensing pathway in our current study and others [31]. Moreover, *LuxS* proteins, which are encoded by the *luxS* gene, were proven to be critical enzymes for activating the bacterial methyl cycle and for producing AI-2 [32], a member of a family of signalling molecules used in quorum sensing. These results indicate that quorum sensing could be associated with the difference in feed efficiency between dairy cows with divergent RFI values. Additionally, the bacterial species showing positive associations with carbohydrate metabolism were all positively correlated with quorum sensing pathways. In short, several *Lachnospiraceae* species, including *Ruminococcus torques*, *Blautia obeum*, *Blautia producta*, *Blautia schinkii*, *Blautia wexlerae, Dorea longicatena, Clostridium clostridioforme, Clostridium symbiosum, Marvinbryantia formatexigens* and *Lachnospiraceae bacterium FE2018,* may play a vital role in both carbohydrate metabolism and quorum sensing. Further studies are required to fully elucidate the relationship between the key microbes and pathways described above to validate our current findings.

## Conclusions

The current study revealed the taxonomic features and functions of rumen microbiomes. Although carbohydrate degradation functions were more abundant in HRFI cows (low efficiency), the higher abundances of butyrate-producing species, functions in butyrate metabolism, methanogenic archaea and methanogenesis pathways indicate that HRFI cows may promote methane production because of their tendency towards butyrate production. Compared with HRFI group, the higher abundances of KO genes related to propionate production and higher tendency of the molar proportion of propionate in LRFI cows may partly explain the higher efficiency in these cows. Several *Lachnospiraceae* species associated with carbohydrate metabolism and quorum sensing may contribute to the differences in RFI. This study provides a in-depth understanding of the microbial ecology of dairy cows with different RFI, and highlights the possibility of modulating the rumen microbiome or microbial functions to improve the feed efficiency of dairy cows. Future studies are required to assess information on the metabolic intermediates related to carbohydrate metabolism, which will provide evidence for the effect of microbial carbohydrate metabolism on feed efficiency.

## Materials and methods

### Experimental design and sample collection

All the procedures involving animals in this study were approved by the Animal Use and Care Committee of Zhejiang University (Hangzhou, China, No. 12410). The RFI value of each animal was calculated as described previously [33]. Briefly, the RFI was estimated as the difference between expected feed intake and actual feed intake, where the expected feed intake was computed through a multiple linear regression model using the regression of actual feed intake on energy-corrected milk yield, metabolic body weight (BW^0.75^), and average daily gain over the measurement period. Based on previous RFI value calculations [33], the 9 dairy cows with the lowest RFI (LRFI) and 9 with the highest RFI (HRFI) were selected from the cohort of 53 multiparous mid-lactation Holstein dairy cows. Power calculations revealed that our sample size will produce 99.0% power while controlling type I error under 5%, based on a t test of RFI. The RFI coefficients distribution of the LRFI and HRFI groups are depicted in Additional file 1: Fig. S4. Cows were housed in a free-stall barn equipped with electronic recognition feeding system for each cow (Zhenghong Co., Shanghai, China). Cows were allowed free access to a total mixed ration consisting of 41% roughage and 59% concentrate, and diet composition has been described previously [32]. Rumen fluid was collected using oral stomach tubes before morning feeding as described by Shen et al. [34]. The pH was immediately measured using a calibrated pH meter (Starter 300; Ohaus Instruments Co. Ltd., Nanjing, China). Samples were stored at − 80 °C until further processing. Two mL of rumen fluid was mixed with 20 μL of 25% orthophosphate acid and then centrifuged at 20,000 × g at 4 °C for 10 min. The supernatant was harvested to use for analysis of VFA by gas chromatograph (GC-2010, Shimadzu, Kyoto, Japan) [35]. Mathematical model was employed to predict methane production by the equation of Ellis et al. [36]: CH_4_ (MJ/d) = 3.23 (± 1.12) + 0.81 (± 0.086) × DMI (kg/d).

### DNA extraction and metagenome sequencing

Microbial DNA was extracted from rumen fluid samples by the repeat bead-beating plus column method [37]. The DNA concentration and purity were determined by using NanoDrop spectrophotometer (Thermo Fisher Scientific, Wilmington, DE, USA). The paired-end library was constructed using TruSeq™ DNA Sample Prep Kit (Illumina, San Diego, CA, USA) and paired-end sequencing was performed on the Illumina HiSeq 4000 platform (150 bp paired-end).

The 3’ and 5’ends were then stripped with SeqPrep, and low-quality reads (length < 50 bp, quality value < 20, or N bases) were removed with Sickle (version 1.33). The reads were aligned to the bovine genome (bosTau8 3.7) with BWA (Version 0.7.9a), and any hit that associated with a read with a corresponding reads was removed [38]. Reads from metagenomic trimmed data were assembled by multiple megahit using Megahit (Version 1.1.2) [39]. Open reading frames (ORFs) in each metagenomic sample were predicted with MetaGene, and the predicted ORFs with lengths > 100 bp were extracted and translated to amino acid sequences using the NCBI translation table [40]. The assembled contigs were clustered using CD-HIT, and non-redundant gene catalogue were constructed based on the sequences from gene sets with a 95% identity (90% coverage) [41]. After quality control, SOAPaligner (Version 2.2.1) was used to map the reads to predicted genes with 95% identity, and the gene abundance in each sample was evaluated [42].

### Taxonomic and functional annotation of rumen microbiota

We used BLASTP (version 2.2.28 +) for taxonomic annotation [43], by aligning the nonredundant gene catalogues against the NCBI NR database [44]. The PCoA based on Bray–Curtis dissimilarity matrices was applied to visualize the taxonomic composition at species level. Microbial taxa with a relative abundance greater than 0.1% in at least 50% of animals within each efficiency group were used for downstream analysis. The KEGG pathway annotation was performed with Diamond against the KEGG database, with an e-value of 1e-5 [45]. Carbohydrate-active enzyme annotation was made with hmmscan (Version 3.1b2). The abundances of pathways, KEGG Orthology (KO), KEGG enzymes, and CAZymes were normalized into counts per million reads (cpm) for downstream analysis.

### Correlation analyses between rumen microbiota and pathways

Correlations between significantly different species and significantly different metabolic pathways were analysed using Spearman’s rank correlation, and significant correlation coefficients with R > 0.5 or < -0.5 (*P* < 0.05) were determined to generate the correlation network. The correlation networks were visualized by Gephi software (version 0.9.2).

### Statistical analysis

Lactation performance and rumen VFAs concentrations were analysed by Student’s t test. Taxonomic and functional data were analysed on the online platform of the Majorbio Cloud Platform [46]. Differential abundances of phylum, family, genus, and CAZymes were tested by Wilcoxon test using stats R package in R software (version 3.3.1). Significance was considered at *P* ≤ 0.05, and a tendency was defined as 0.05 < *P* ≤ 0.10.

## Supplementary Information

**Additional file 1: Table S1**. Comparison of lactation performance between the cows with high (HRFI) and low residual feed intake (LRFI). **Table S2**. Summary of sequence data generated from rumen samples of 9 high (H) and 9 low (L) residual feed intake cows. **Table S3**. Permutational multivariate analysis of variance (PERMANOVA) for the Bray–Curtis dissimilarity matrices for microbial taxonomy between the cows with high (HRFI) and low residual feed intake (LRFI). **Figure S1**. Rumen microbial composition based on the domain level taxonomy. **Figure S2**. Microbial profiles of the cows with high (HRFI) and low residual feed intake (LRFI). **Figure S3**. Microbial metabolic pathways and carbohydrate-active enzymes (CAZymes) of the cows with high (HRFI) and low residual feed intake (LRFI). **Figure S4**. Distribution of residual feed intake in high (HRFI) and low residual feed intake (LRFI) cows.

## Data Availability

The Illumina sequencing raw data for our samples have been deposited in the NCBI Sequence Read Archive (SRA) under accession numbers: PRJNA597489.
