# Supplementary material for: Metagenomics reveals differences in microbial composition and metabolic functions in the rumen of dairy cows with different residual feed intake
Source: Anim Microbiome. 2022 Mar 8;4:19. doi: 10.1186/s42523-022-00170-3 (PMC8902708; doi:10.1186/s42523-022-00170-3)
Supplement: Supplementary file 1 — Additional file 1: Table S1. Comparison of lactation performance between the cows with high (HRFI) and low residual feed intake (LRFI). Table S2. Summary of sequence data generated from rumen samples of 9 high (H) and 9 low (L) residual feed intake cows. Table S3. Permutational multivariate analysis of variance (PERMANOVA) for the Bray–Curtis dissimilarity matrices for microbial taxonomy between the cows with high (HRFI) and low residual feed intake (LRFI). Figure S1. Rumen microbial composition based on the domain level taxonomy. Figure S2. Microbial profiles of the cows with high (HRFI) and low residual feed intake (LRFI). Figure S3. Microbial metabolic pathways and carbohydrate-active enzymes (CAZymes) of the cows with high (HRFI) and low residual feed intake (LRFI). Figure S4. Distribution of residual feed intake in high (HRFI) and low residual feed intake (LRFI) cows. [file 42523_2022_170_MOESM1_ESM.docx]

**Supplementary Information**

**Table S1.** Comparison of lactation performance between the cows with low and high residual feed intake (RFI).

| Items | Low RFI | High RFI | SEM | *P* value |
| --- | --- | --- | --- | --- |
| DMI^1^, kg/d | 25.5 | 28.0 | 0.76 | 0.04 |
| Milk yield, kg/d |  |  |  |  |
| Milk | 37.5 | 35.1 | 1.81 | 0.37 |
| ECM^2^ | 40.0 | 39.2 | 2.01 | 0.77 |
| Milk components |  |  |  |  |
| Fat, kg/d | 1.50 | 1.48 | 0.09 | 0.90 |
| Fat, % | 4.01 | 4.24 | 0.18 | 0.39 |
| Protein, kg/d | 1.21 | 1.21 | 0.05 | 0.99 |
| Protein, % | 3.24 | 3.44 | 0.06 | 0.04 |
| Lactose, kg/d | 1.91 | 1.74 | 0.09 | 0.22 |
| Lactose, % | 5.09 | 4.95 | 0.04 | 0.05 |
| Feed efficiency |  |  |  |  |
| Milk/DMI, kg/kg | 1.46 | 1.25 | 0.05 | <0.01 |
| ECM/DMI, kg/kg | 1.56 | 1.40 | 0.05 | 0.03 |
| RFI, kg/d | -1.48 | 1.51 | 0.16 | <0.01 |

^1^ DMI, dry matter intake;

^2^ECM (kg) = 0.3246 × milk yield (kg) + 13.86 × milk fat (kg) + 7.04 × milk protein (kg).

**Table S2.** Summary of sequence data generated from the rumen samples of 9 high (H) and 9 low (L) residual feed intake cows.

| Sample | Raw reads | Reads after QC | Clean reads | Contigs | N50(bp) | ORFs |
| --- | --- | --- | --- | --- | --- | --- |
| H1 | 50,945,870 | 50,580,246 | 50,573,618 | 706,438 | 639 | 973,244 |
| H2 | 56,101,136 | 55,549,854 | 55,520,580 | 900,945 | 671 | 1,221,276 |
| H3 | 51,514,982 | 51,093,030 | 51,085,230 | 547,088 | 726 | 763,118 |
| H4 | 55,151,200 | 54,709,852 | 54,702,046 | 625,744 | 771 | 894,454 |
| H5 | 50,440,260 | 50,095,884 | 50,042,796 | 670,234 | 699 | 920,849 |
| H6 | 55,731,048 | 55,319,794 | 55,308,920 | 850,402 | 684 | 1,151,898 |
| H7 | 50,511,818 | 50,031,306 | 49,993,862 | 778,927 | 698 | 1,060,931 |
| H8 | 47,122,882 | 46,671,848 | 46,661,512 | 412,429 | 794 | 584,260 |
| H9 | 53,746,534 | 53,348,072 | 53,341,586 | 780,320 | 690 | 1,070,114 |
| L1 | 45,422,276 | 45,040,430 | 45,034,678 | 402,639 | 1,073 | 618,819 |
| L2 | 47,417,900 | 47,001,990 | 46,991,972 | 692,383 | 677 | 915,481 |
| L3 | 48,761,068 | 48,391,500 | 48,381,888 | 638,840 | 678 | 844,382 |
| L4 | 46,908,870 | 46,572,922 | 46,561,276 | 688,812 | 697 | 933,449 |
| L5 | 50,479,622 | 50,051,794 | 50,042,500 | 440,760 | 733 | 598,373 |
| L6 | 46,229,640 | 45,838,360 | 45,831,838 | 343,837 | 877 | 499,174 |
| L7 | 49,965,168 | 49,569,116 | 49,556,432 | 657,868 | 675 | 876,801 |
| L8 | 54,137,030 | 53,669,940 | 53,657,832 | 805,794 | 667 | 1,087,668 |
| L9 | 48,507,246 | 48,038,572 | 48,024,966 | 726,551 | 653 | 949,529 |
| Total | 909,094,550 | 901,574,510 | 901,313,532 | 11,670,011 | 13,102 | 15,963,820 |
| mean | 50,505,253 | 50,087,473 | 50,072,974 | 648,334 | 728 | 886,879 |
| SD | 3,347,269 | 3,322,956 | 3,320,999 | 160,922 | 103 | 205,231 |
| SEM | 928,365 | 921,622 | 921,079 | 44,632 | 29 | 56,921 |

**Table S3**. Permutational multivariate analysis of variance (PERMANOVA) for the Bray–Curtis dissimilarity matrices for microbial taxonomy between the cows with high (HRFI) and low residual feed intake (LRFI).

| Taxonomy | SumsOfSqs | MeanSqs | F.Model | R2 | P.adjust |
| --- | --- | --- | --- | --- | --- |
| Bacteria | 0.012 | 0.012 | 1.906 | 0.106 | 0.166 |
| Eukaryota | 0.199 | 0.199 | 1.363 | 0.078 | 0.253 |
| Archaea | 0.071 | 0.071 | 2.126 | 0.117 | 0.145 |
| Viruses | 0.075 | 0.075 | 2.252 | 0.123 | 0.134 |


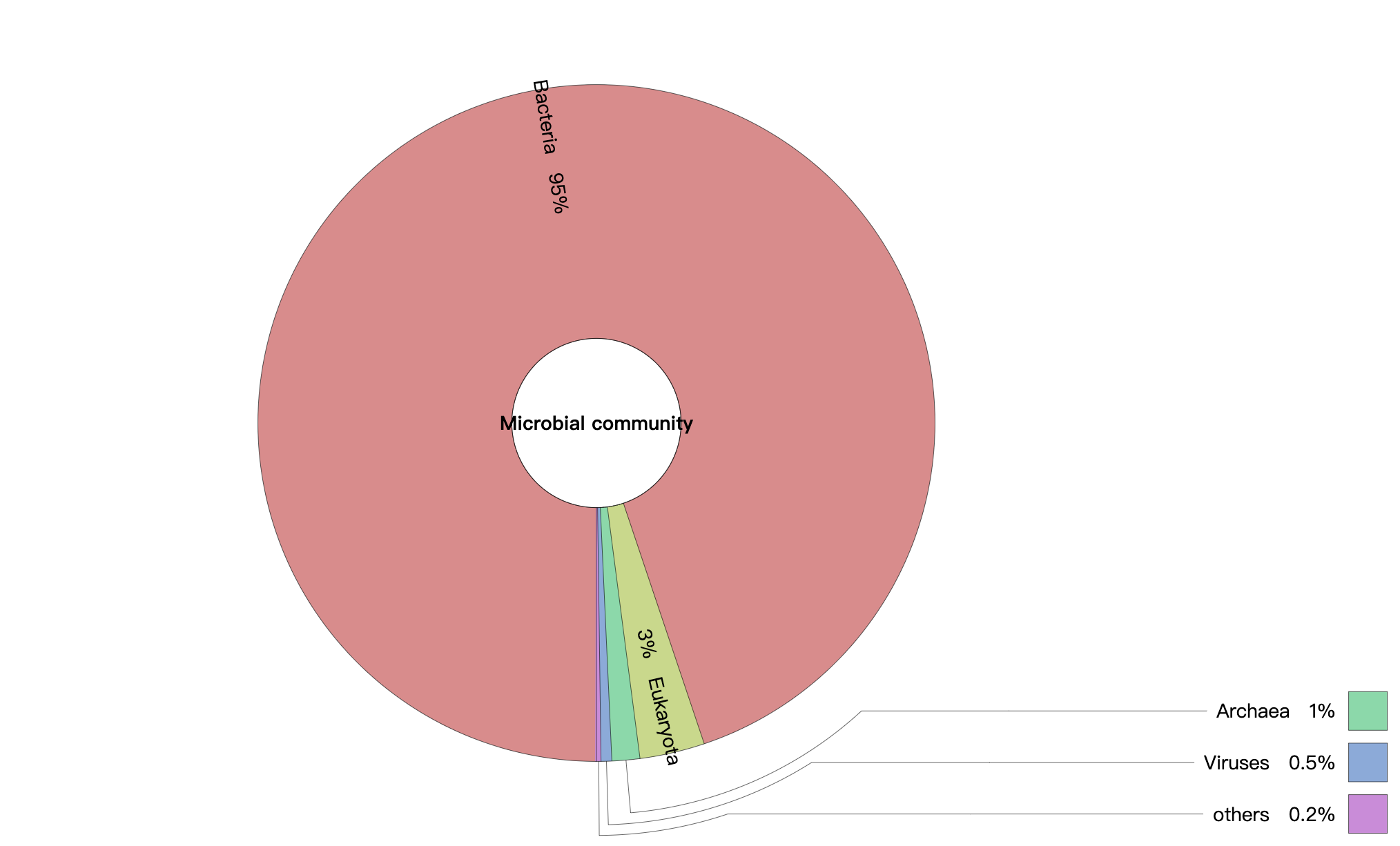


**Figure S1**. Rumen microbial composition based on the domain level taxonomy.

**Figure S2**. Microbial profiles of the cows with high (HRFI) and low residual feed intake (LRFI). (A) Archaeal compositional profiles of HRFI and LRFI cows’ rumen samples based on species visualized using PCoA. (B) Bacterial profiles of HRFI and LRFI cows’ rumen samples based on species visualized using PCoA.


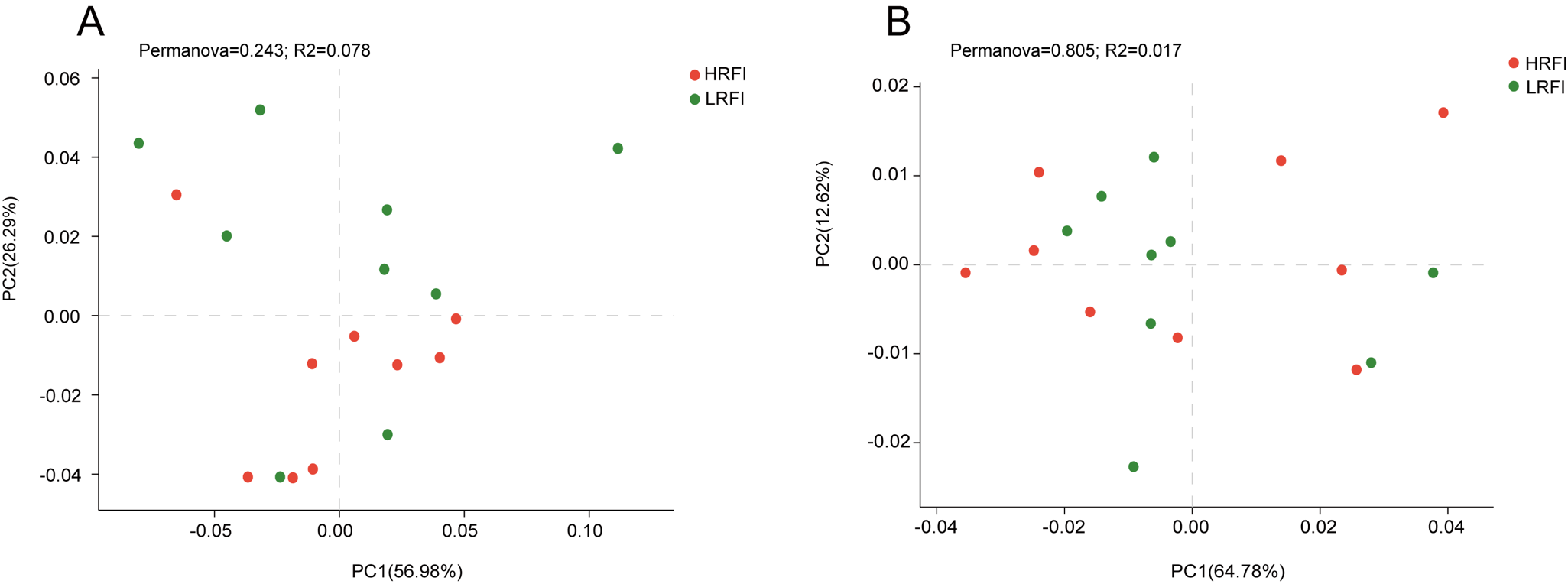


**Figure S3**. Microbial metabolic pathways and carbohydrate-active enzymes (CAZymes) of the cows with high (HRFI) and low residual feed intake (LRFI). (A) PCoA based on microbial metabolic pathways. (B) PCoA based on CAZymes.

**Figure S4.** Distribution of residual feed intake in two feed efficiency groups. HRFI = High residual feed intake; LRFI = Lou residual feed intake.
